# Birth order is associated with caries development in young children: a register-based cohort study

**DOI:** 10.1186/s12889-020-8234-7

**Published:** 2020-02-12

**Authors:** A. Julihn, F. C. Soares, U. Hammarfjord, A. Hjern, G. Dahllöf

**Affiliations:** 1 Division of Orthodontics and Pediatric Dentistry, Department of Dental Medicine, Karolinska Institutet, Stockholm, Sweden; 2 Department of Pediatric Dentistry, Eastman Institute, Public Dental Service, Stockholm, Sweden; 3 Center for Pediatric Oral Health Research, Stockholm, Sweden; 4 Public Dental Service Gothenburg, Västra Götaland, Sweden; 5 Clinical Epidemiology, Department of Medicine, Karolinska Institutet and Centre for Health Equity Studies (CHESS), Stockholm, Sweden; 6 Center for Oral Health Services and Research, Mid-Norway, TkMidt, Trondheim, Norway

**Keywords:** Birth rank, Cohort study, Dental caries, Epidemiology, Preschool children, Register-based, Risk assessment

## Abstract

**Background:**

Birth order has been shown to affect the health of the child; less is known, however, about how birth order affects caries development in children. Thus, the present study investigated the association between birth order and dental caries development in young children.

**Methods:**

This retrospective registry-based cohort study included all children born in 2000–2003 who were residing in Stockholm County, Sweden, at age 3 years (*n* = 83,147). The study followed the cohort until subjects reached 7 years of age. Children with registry data on dental examinations and sociodemographic characteristics at ages 3- and 7 years constituted the final study cohort (*n* = 65,259). The outcome variable was “caries increment from age 3- to 7 years” (Δdeft > 0) and the key exposure, “birth order”, was divided into five groups. A forward stepwise logistic binary regression was done for the multivariate analysis with adjustments for sociodemographic factors.

**Results:**

At age 3 years, 94% had no fillings or manifest caries lesions. During the study period, 22.5% (*n* = 14,711) developed dental caries. The final logistic regression analysis found a statistically significant positive association between birth order and caries increment. Further, excess risk increased with higher birth order; with the mother’s first-born child as reference, risk for the second-born child was OR 1.17, 95% CI = 1.12–1.23; for the third-born child, OR 1.47, 95% CI = 1.38–1.56; for the fourth-born child, OR 1.69, 95% CI = 1.52–1.88; and for the fifth-born or higher birth-order child, OR 1.84, 95% CI = 1.58–2.14.

**Conclusions:**

These findings show that birth order influences caries development in siblings, suggesting that birth order can be regarded as a predictor for caries development in young children. This factor may be helpful in assessing caries risk in preschool children and should be considered in caries prevention work in young children with older siblings.

## Background

Previous research has shown that birth order affects health. First-born children are reported to have lower birth weight and are more likely to be hospitalized for perinatal conditions and congenital malformations in early childhood compared to their later-born siblings [1, 2]. However, the disadvantage of older siblings is reversed as they grow older since later-born children in large families are reported to be at an increased risk of hospitalization for injuries and avoidable conditions, which is possibly related to less parental attention [1, 3]. Moreover, risk of earlier death for later-born siblings is higher throughout adulthood [4].

Poor health is strongly correlated with socioeconomic background and is transmitted across generations [5, 6]. The differences in health between siblings in a family could be due to many factors, such as parental time and changes in the family environment due to the presence of children of different ages. The first-born child does not share parental time with any siblings, at least not during the first period in life. Since parental time is finite, succeeding children receive less quality time during their first years [7, 8].

Literature on the association between birth order and children’s oral health is scarce. The few studies in this area are small, and the subgroups of birth order vary. Although risk of dental caries in later-born children compared to first-born children has been found to be higher [9, 10], conflicting results have also been reported [11, 12].

Thus, our objective was to study dental caries development between age 3 and 7 years in relation to birth order in a large Swedish cohort. The hypothesis was that children with a higher birth order develop more caries.

## Methods

### Study design

The present study is a retrospective, registry-based cohort study using information collected from data sources at the Public Health Care Administration in Stockholm as well as from national registries at the National Board of Health and Welfare and at Statistics Sweden (SCB). The Regional Ethics Board in Stockholm and the Swedish Data Inspection Board, a national agency that serves as an institutional review board for studies using database linkage, approved the protocol for this study.

### Subjects

The study included all children born in 2000–2003 who resided in Stockholm County, Sweden at age 3 years (*n* = 83,147). We followed this cohort until the individuals reached age 7 years. During this period the subjects received regular dental check-ups at the Public Dental Service, with private practitioners, or at the Division of Pediatric Dentistry, Department of Dental Medicine, Karolinska Institutet. The final study cohort comprised 65,259 children (33,423 boys and 31,836 girls) who had been examined at both 3- and 7 years of age. The drop-out rate was 22%, and the most common reason for dropping out was that the child had moved out of the area. The sample calculation was carried out a posteriori, and for the analysis conducted in the present study, it is possible to detect as significant odds ratio higher than 1.2, with 95% confidence interval, and statistical power higher than 80%.

### Collection of dental caries data

Data on manifest caries lesions were collected from clinical and radiographic examinations as decayed teeth (dt), extracted teeth (et), and filled teeth (ft). Manifest caries was defined as caries on smooth surfaces at the lowest level that can be verified as a cavity and is detectable by probing or, in fissures, by a catch of the probe under slight pressure. Proximal caries on radiographs was defined as manifest caries in which the lesion clearly extends into the dentin [13]. Only children with clinical indications received a radiographic examination. The decayed, extracted, and filled primary teeth (deft) index was then calculated to determine the severity of the caries experience at ages 3- and 7 years. Caries increment from age 3- to 7 years comprised all new caries lesions in primary teeth (i.e., the difference between the deft values at age 3- and 7 years). No permanent teeth were included in the 7-year outcomes.

### Population-based registries

In Sweden, the personal identity number (PIN), a 10-digit number unique for each resident and used for indexing in all health and census registries, makes the national registries extremely useful in epidemiological research [14]. Information on individuals is easily extracted from the various registries. The present study used information from the Medical Birth Registry (MBR), the Total Population Registry (TPR), the Income and Taxation Registry (IoT), and the Registry of Education.

### Medical birth registry

The Centre for Epidemiology at the Swedish National Board of Health and Welfare maintains the Swedish Medical Birth Registry (MBR). We collected the following information from the MBR: gender, birth order, maternal age, family situation, maternal smoking habits during early pregnancy, and the mother’s height and weight at the first visit to the public maternity healthcare clinic. We chose “birth order” as our key exposure. Due to their low number, we merged families with five or more children into one group; thus, “birth order” had five subgroups: first-born, second-born, third-born, fourth-born, and fifth- or later-born children. Maternal age was categorized into three subgroups: < 25 years, 25–34 years, and ≥ 35 years. Family situation was dichotomized into cohabiting or single parents. Smoking habits during early pregnancy were dichotomized into no or daily smoking. Body mass index (BMI) of the mother was calculated and classified as BMI < 25.00, BMI = 25.00–29.99 and BMI ≥ 30.00. Table 2 presents all collected child and parental characteristics.

### Total population registry

The SCB maintains the TPR. We collected data on maternal and paternal country of birth from the TPR and dichotomized birth country as “Sweden” or “abroad” for the mother and the father.

### Income and taxation registry

The Swedish National Tax Board collects data on individuals’ annual income tax based on income tax returns and tax-authority decisions. The Board then sends summary statistics to the SCB. From this registry, we collected information regarding the family’s disposable income from the 2004 survey. We categorized and analyzed the variable “family income” into five quintiles from highest to lowest income in the statistical analysis.

### Registry of education

We obtained data on maternal education level from the Registry of Education, which is updated each year in April. In the statistical analysis, we classified “education level” according to the number of years of schooling in 2004 as low (≤ 9 years), intermediate (10–12 years), and high (≥ 13 years), (Table 2).

### Statistical analysis

The Statistical Package for the Social Sciences (IBM SPSS® Statistics version 22.0, [SPSS, Inc., IBM Corp., Armonk, NY, USA]) and STATA 13 for Windows (StataCorp. 2013. *Stata Statistical Software: Release 13*. College Station, TX: StataCorp LP) was used for data analysis. Descriptive analyses included relative and absolute frequencies, means, and standard deviations. Differences between categorical variables were assessed using the chi-square test.

Forward stepwise binary logistic regression was done to analyze the associated factors of the outcome “caries increment from age 3- to 7 years” (Δ deft > 0). The five subgroups of “birth order”, the key exposure, were analyzed in an univariate analysis (Table 2) and a multivariate analysis (Table 3). The analyses dichotomized all outcomes to distinguish between subjects with and without caries increment (Δ deft > 0 or = 0, respectively).

The final regression models were adjusted for potential confounders. A combination of methods was used to select the confounders included in the models; confounders were selected based on their association with the outcome, as well as based on their association with the key exposure and their subsequent influence on the outcome [6]. Model I was adjusted for gender and maternal age; Model II, for maternal smoking and maternal BMI; and Model III, for socio-demographic factors (parent’s country of birth, maternal educational level, family situation, and family income). Model IV included all groups of variables as adjusted. The confounders are aligned with factors in conceptual models of caries development [15]. The odds ratios (OR) with a 95% CI and statistical significance set at α = 0.05 was used to estimate associations.

## Results

**Fig. 1:**
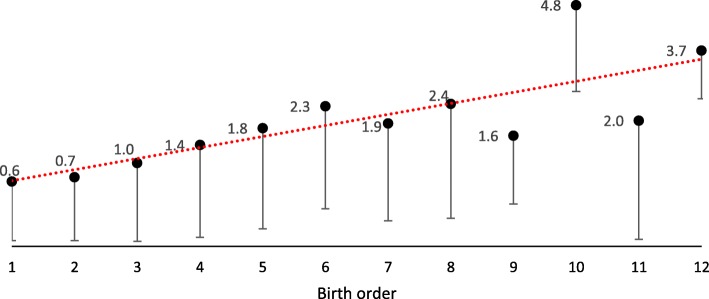
Mean and standard deviation of teeth with caries experience at 7 years according to birth order. Number of children with “birth order 1” = 30,057 children; “2” = 23,028 children; “3” = 8021 children; “4” = 1971 children; “5” = 571 children; “6” = 161 children; “7” = 58 children; “8” = 45 children; “9” = 11 children; “10” = 4 children; “11” = 4 children; and “12” = 3 children

In the final cohort of 65,259 children, 51% were boys (33,423). Forty-seven percent (30,524) were first-born children, 36% (23,488) second-born, 13% (8250) third-born, 3% (2063) fourth-born, and 1% (934) fifth or later-born children (Table 1). At age 3 years, 94% had no fillings or manifest caries lesions. During the study period, 22.5% (14,711) developed dental caries and at age 7 years, 75% were still caries-free. The mean deft index at age 3 years was 0.16 ± 0.80 and at age 7 years, 0.73 ± 1.62. The mean deft at age 3 was among first-born children 0.12 ± 0.67, second-born children 0.14 ± 0.75, third-born children 0.24 ± 0.98, fourth-born children 0.43 ± 1.35 and fifth or later-born children 0.68 ± 1.67. Figure 1 presents the mean of deft at age 7 years in relation to birth order. Among first-born children, mean deft was 0.6 ± 1.4, while the mean deft of children who was the last-born of twelve children was 3.7 ± 1.2.

**Table 1:** Child and family characteristics

	n	%
Gender		
Boy	33,423	51.2
Girl	31,836	48.8
Birth order		
First-born	30,524	46.8
Second-born	23,488	36.0
Third-born	8250	12.6
Fourth-born	2063	3.2
Fifth- or later-born	934	1.4
Maternal country of birth		
Sweden	50,110	76.8
Abroad	15,146	23.2
Paternal country of birth		
Sweden	49,042	75.5
Abroad	15,909	24.5
Maternal education		
≤ 9 years	5593	8.6
10–12 years	25,603	39.2
≥ 13 years	34,063	52.2
Family situation		
Cohabiting	62,298	95.5
Single	2961	4.5
Family income		
First quintile (highest income)	13,427	20.7
Second quintile	13,303	20.5
Third quintile	13,204	20.3
Fourth quintile	12,810	19.7
Fifth quintile (lowest income)	12,168	18.7
Social welfare allowance		
No	63,051	97.1
Yes	1861	2.9
Maternal smoking in early pregnancy		
No	55,352	84.8
Daily	3616	5.6
Missing	6291	9.6
Maternal BMI in early pregnancy		
< 25.00	45,490	69.7
25.00**–**29.99	10,474	16.0
≥ 30.00	3789	5.8
Missing	5506	8.5
Maternal age at delivery		
25–34 years	43,138	66.1
< 25 years	6561	10.1
≥ 35 years	15,560	23.8

**Fig. 2:**
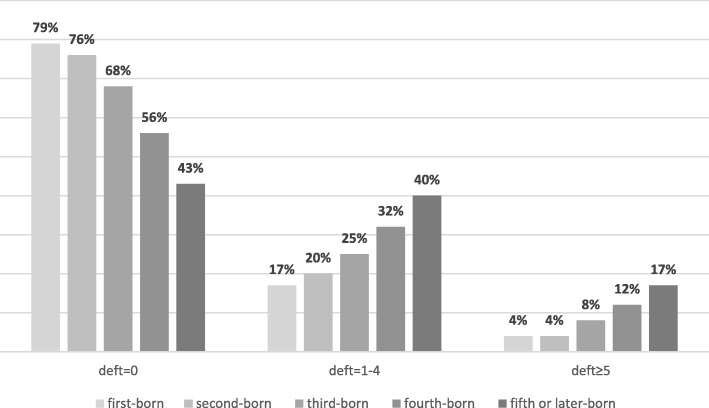
Proportion of children with caries experience at 7 years in relation to birth order

Table 2 presents the association between caries increment (age 3–7 years) and child, maternal, and family factors. In this crude analysis, all background factors were significantly associated with caries increment. The probability of caries increment increased with birth order. The proportion of children who were caries free at age 7 years was highest among first-born children (79%), decreasing to 43% in fifth-born or higher. Similarly, the proportion of children with caries increased with increasing birth order (Fig. 2).

**Table 2:** Characteristics of children with caries increment from age 3- to 7 years and of their parents

Variables	Caries increment (Δ deft = 0)	Caries increment (Δ deft > 0)
	(*n* = 50,548)	(*n* = 14,711)
	%	% P^a^
Gender		
Boy	77	23 < 0.001
Girl	78	22
Birth order		
First-born	81	19 < 0.001
Second-born	78	22
Third-born	71	29
Fourth-born	61	39
Fifth- or later-born	52	48
Maternal country of birth		
Sweden	83	17 < 0.001
Abroad	60	40
Paternal country of birth		
Sweden	83	17 < 0.001
Abroad	61	39
Maternal education level		
≥ 13 years	83	17 < 0.001
10–12 years	74	26
≤ 9 years	57	43
Family situation		
Cohabiting	78	22 < 0.001
Single	66	34
Family income		
First quintile (highest)	85	15 < 0.001
Second quintile	83	17
Third quintile	80	20
Fourth quintile	75	24
Fifth quintile (lowest)	64	36
Social welfare allowance		
No	78	22 < 0.001
Yes	55	45
Maternal smoking in early pregnancy		
No	86	14 < 0.001
Yes	81	19
Maternal BMI in early pregnancy		
< 25.00	79	21 < 0.001
25.00**–**29.99	75	25
≥ 30.00	70	30
Maternal age at delivery		
25–34 years	79	21 < 0.001
< 25 years	64	36
≥ 35 years	78	22

^a^*P*-values refer to univariate analyses in a binary logistic regression model

Table 3 presents the results of a multivariate logistic regression analysis of the associations between birth order and caries increment from age 3- to 7 years. The analysis was adjusted for potential confounding factors in four different models. Independent of adjustments, all subgroups of birth order were significantly associated with caries increment from age 3- to 7 years. Model IV included all groups of potential confounders and found that, compared with the first-born child, second-born children had 1.17 times higher odds of having a caries increment from age 3- to 7 years (1.17 [1.12–1.23]); third-born, 1.47 times higher odds (1.47 [1.38–1.56]); fourth-born, 1.69 times higher odds (1.69 [1.52–1.88]); and fifth-born and higher birth-order children, 1.84 times higher odds (1.84 [1.58–2.14]).

**Table 3:** Multivariate logistic regression with key exposure birth order and caries increment from age 3- to 7 years

Birth order	Caries increment (Δ deft > 0)				
	Crude Model	Model I	Model II	Model III	Model IV
	OR (CI 95%)	OR (CI 95%)	OR (CI 95%)	OR (CI 95%)	OR (CI 95%)
First-born	1.00	1.00	1.00	1.00	1.00
Second-born	1.21 (1.16–1.26)	1.21 (1.16–1.26)	1.20 (1.15–1.26)	1.18 (1.12–1.23)	1.17 (1.12–1.23)
Third-born	1.76 (1.67–1.86)	1.76 (1.66–1.86)	1.71 (1.61–1.81)	1.50 (1.42–1.60)	1.47 (1.38–1.56)
Fourth-born	2.70 (2.46–2.96)	2.69 (2.45–2.95)	2.43 (2.20–2.68)	1.84 (1.67–2.04)	1.69 (1.52–1.88)
Fifth- or later-born	3.92 (3.44–4.47)	3.90 (3.41–4.45)	3.55 (3.08–4.08)	1.93 (1.68–2.23)	1.84 (1.58–2.14)

Crude model = Unadjusted.

Model I = Adjusted only for gender and maternal age.

Model II = Adjusted only for maternal smoking and BMI.

Model III = Adjusted only for sociodemographic factors (parent’s country of birth, maternal educational level, family situation and family income).

Model IV = Adjusted for gender, maternal age, maternal smoking, maternal BMI, and sociodemographic factors.

## Discussion

The findings of this registry-based longitudinal cohort study revealed that birth order is associated with caries increment in young children between age 3 and 7 years. Compared with first-born children, the highest risk of caries increment occurred in fifth- or later-born children. Further, the excess risk of a higher birth order compared to first-born children concerning caries increment was positively correlated with the number of caries lesions during the 4-year study period. Thus, our hypothesis was true.

The general health and oral health status of a child is largely associated with parental characteristics [5, 6, 16–18]. Parental influence on dental health is reported to be stronger for preschool children than older children [19]. To control for possible sociodemographic confounders in our analyses, we used population-based registries that could supply detailed information on parental background characteristics such as maternal age at delivery, maternal education, parental ethnic background, and family situation and income at the start of the follow-up period. We also extracted information on maternal smoking habits and BMI in early pregnancy and made adjustments for these variables in our statistical analyses since both smoking and BMI are associated with childhood caries [20–22]. In this study, the birth order of the child was used as the key exposure. On average, children of higher birth order have older mothers, and mothers with large families have their first child at a younger age. This may bias the estimated effect of birth order. To reduce this bias, we also controlled for maternal age at birth.

The present study is the first to compare succeeding birth orders, up to “fifth-born or later”, with the first-born child’s risk of developing caries. Other studies, however, have investigated the influence of birth order on dental caries in children, but these studies have used small samples and “birth order” is often dichotomized and variably defined.

The novel findings in this study are that second-, third-, fourth-, and fifth- or later-born children have a significantly increased risk of developing new caries lesions between age 3- and 7 year compared with first-born children. Our results are in line with those of Wellappuli and Amarasena [9] who reported that a birth rank > 1 was significantly associated with dental caries experience in 3–5-year-old children compared with children with a birth rank of 1. Recently, studies from Nigeria found more caries in children with three or more siblings compared to 0–2 siblings [10, 23]. Previous studies have reported somewhat contradictory results concerning birth order and family size in relation to caries. In one study, it appeared that children at either birth order extreme (birth rank 1 and greater than 3) were more susceptible to dental caries than children with a birth order of 2 or 3 [11]. Also, caries-free preschool children have been found to occur more frequently at lower birth orders [24]. In contrast to the results in the present study, Wigen and co-authors [12] found no association between the presence of older siblings in the family and caries experience in 5-year-old children in Norway, a country with a public dental care system similar to the one in Sweden, albeit with a more homogenous population concerning ethnic background. The finding that the child’s ordinal position in the family was directly proportional to the risk of caries increment from age 3- to 7 years is interesting and agrees with Chung and co-authors [25], who demonstrated a positive correlation between increasing ordinal position in the family and caries prevalence in 12–18-year-old children.

Family size has also been associated with an increased risk of caries experience in preschool children [9, 18, 26], but the influence of family size upon dental caries in preschool children is also conflicting. The present study, however, does not analyze variable family size since we only retrieved data at the time of the child’s birth.

The family system that surrounds a child’s home environment will play an active role in establishing and promoting behaviors, such as oral hygiene practices and dietary habits [5, 18, 27, 28], that will persist throughout life [29, 30]. We speculate that when the number of children in a family is high, the individual care and attention given by parents to a child may be less. Our assumption rests on the resource dilution hypothesis proposed by Blake [7] and elaborated by Downey [31]. The resource dilution hypothesis proposes that sibling variables, such as number of children in a family and the birth order position of children, are related to the cultural and material resources that parents provide for their children. The greater the number of children in a family or the later their birth order position, the more they have to share family resources and the lower their performance [32]. In most countries, the chances to obtain a college degree are negatively associated with family size [33]. Asano [34] found that psychological support from parents reported by the eldest siblings is significantly higher than for the youngest. Studies have observed that earlier-born children are subject to more rules and monitoring by parents than later-born children [35, 36]. Other hypotheses regarding the mechanisms behind the birth-order effect suggest sibling influences [37], and strategic parental behavior [36]. In line with this, we speculate that later-born children are likely to be exposed to sugar-containing products earlier compared with first-born children. Regarding sugar introduction, dietary patterns in infancy, characterized by a greater number of highly sweetened foods and drinks in the first year of age, are strongly associated with the incidence of childhood caries in subsequent years [38]. However, empirical evidence on which mechanisms are most important for explaining birth-order effects is limited. Our results suggest that differential parental investments may partially explain birth-order effects because parental time and resources are limited, and thus, the risk of developing dental caries is higher.

The psychosocial factors affecting parental ability to maintain the oral health of their children might be reflected in several variables in the family structure. Another plausible mechanism is that, on average, later-born children are more exposed to family disruptions, experiencing events, such as divorce and loss of a parent, at younger ages than their older siblings [39].

Compared with other registry-based research studies, our study has inherent limitations. No information on dietary or oral hygiene habits could be retrieved, since this information are not included in Swedish health and oral health registers.

Random errors pose a risk because the diagnosis of manifest caries is occasionally under- or over-reported due to inter-variability. However, increasing the size of the cohort reduces random errors, which approach zero if the cohort becomes infinitely large [40]. In the present study, the final cohort comprised 65,259 children, so the risk of random error is minor. Our results can be considered generalizable to Swedish preschool children since all children born in 2000–2003 who were residing in Stockholm County, Sweden, at age 3 years and were followed until age 7 years were included. The drop-out rate of 22% is another limitation. Children who were lost to follow-up generally came from families with a significantly lower socioeconomic status, that is, lower educational level and lower family income. Furthermore, the mothers of dropouts were more often born abroad, smoked in early pregnancy, and obese. However, in this study, the most common reason for dropping out was that the child had moved out of the area and no significant differences in child characteristics between the dropouts and the study population were observed.

## Conclusions

The present study demonstrates how later-born children are at risk of developing caries between age 3- and 7 years compared to first-born children. Since risk increases with higher birth order, this information should be helpful for pediatric dental personnel when assessing caries risk in young children with older siblings, thus ensuring that parents of large families receive adequate guidelines and the young children, the maximum benefit of the various available preventive systems.

## Data Availability

The data that support the findings of this study is available from the Swedish National Board and Welfare but restrictions apply to the availability of these data, which were used under license for the current study, and so are not publicly available. Questions or requests concerning this data is directed to the principal investigator Annika Julihn.

## Abbreviations

BMI: Body Mass Index
CI: Confidence Interval
deft: Decayed, extracted, filled teeth
IoT: The Income and Taxation Registry
MBR: Medical Birth Registry
OR: Odds ratio
PIN: Personal Identity Number
SCB: Statistics Sweden
TPR: The Total Population Registry
